# Artificial whole genome duplication in paleopolyploid sturgeons yields highest documented chromosome number in vertebrates

**DOI:** 10.1038/s41598-020-76680-4

**Published:** 2020-11-12

**Authors:** Ievgen Lebeda, Petr Ráb, Zuzana Majtánová, Martin Flajšhans

**Affiliations:** 1 Faculty of Fisheries and Protection of Waters, South Bohemian Research Center of Aquaculture and Biodiversity of Hydrocenoses, Research Institute of Fish Culture and Hydrobiology, University of South Bohemia in České Budějovice, Zátiší 728/II, 389 25 Vodňany, Czech Republic; 2 Laboratory of Fish Genetics, Institute of Animal Physiology and Genetics, Czech Academy of Sciences, Rumburská 89, 277 21 Liběchov, Czech Republic

**Keywords:** Agricultural genetics, Cytogenetics

## Abstract

Critically endangered sturgeons, having undergone three whole genome duplication events, represent an exceptional example of ploidy plasticity in vertebrates. Three extant ploidy groups, combined with autopolyploidization, interspecific hybridization and the fertility of hybrids are important issues in sturgeon conservation and aquaculture. Here we demonstrate that the sturgeon genome can undergo numerous alterations of ploidy without severe physiological consequences, producing progeny with a range of ploidy levels and extremely high chromosome numbers. Artificial suppression of the first mitotic division alone, or in combination with suppression of the second meiotic division of functionally tetraploid zygotes (4n, C-value = 4.15) of Siberian sturgeon *Acipenser baerii* and Russian sturgeon *A. gueldenstaedtii* resulted in progeny of various ploidy levels—diploid/hexaploid (2n/6n) mosaics, hexaploid, octoploid juveniles (8n), and dodecaploid (12n) larvae. Counts between 477 to 520 chromosomes in octoploid juveniles of both sturgeons confirmed the modal chromosome numbers of parental species had been doubled. This exceeds the highest previously documented chromosome count among vertebrates 2n ~ 446 in the cyprinid fish *Ptychobarbus dipogon.*

## Introduction

Polyploidization refers to the multiplication of one or more complete chromosome sets in an organism and represents an important step in evolution and speciation. Polyploidy provides beneficial genetic flexibility and broad adaptive responses, i.e., additional gene copies theoretically allow evolution under reduced selective constraint and the acquisition of novel gene functions that contribute to adaptation^1^. Occurrence of polyploidy in vertebrates is rather sporadic compared to invertebrates and plants, and is mostly restricted to amphibians and ray-finned fishes^2,3^. Ancestral vertebrate genomes likely underwent two rounds (1R and 2R) of whole genome duplication (WGD)^4–6^. Teleost fishes then underwent a teleost-specific round of WGD (3R or TSGD)^7,8^ which is possibly responsible for the great diversity of this group, forming about half of extant vertebrate species^9^. Additional lineage-specific WGD events have occurred independently in several teleostean lineages, e.g., 4R in Botiidae^10^, Catostomidae^11^, Cyprinidae^12,13^, Callichthyidae^14^ and Salmonidae^15^. These duplication events were associated with increased chromosome numbers from prevailing teleost 2n = 48–50^16^ to roughly (paleo)4n = 90–104^17^. However, in several lineages of Cyprinidae the already increased 2n has been raised to around (paleo)6n = 150, i.e., a duplication of a 3n derivative of the previous (paleo)4n level^16^. The highest chromosome count of any vertebrate to date was documented in *Ptychobarbus dipogon,* a representative of the schizothoracine cyprinid lineage (Cypriniformes) from the Tibetan Plateau. It possesses 446 chromosomes, representing (paleo)3n derivative of already (paleo)6n^18^.

Four basal clades of non-teleostean fishes, i.e., bichirs (Polypteriformes), gars (Lepisosteiformes), bowfin (Amiiformes) and sturgeons and paddlefishes (Acipenseriformes) diverged from the teleosts long before the TSGD^19–22^. With the exception of Acipenseriformes, none of these ancient lineages underwent further WGD^23^. Of the Acipenseriformes, the Acipenserids (sturgeons) went through up to three rounds of lineage specific WGDs^24,25^, whereas the Polyodontids (paddlefishes) went through only one^26^. The first occurred in the most recent common ancestor of sturgeon and paddlefish with 2n = 60^27^ leading to a 2n =  ~ 120 chromosome lineage of 180 (124–225) Ma age^28^. The second lineage specific WGD took place separately in each sturgeon lineage leading to two ~ 240-chromosome clades^25^ in the Atlantic (~ 53 Mya) and Pacific (~ 70 Mya) lineages. The third WGD was unique to the shortnose sturgeon (*Acipenser brevirostrum*) dated ~ 35 Mya^25^ and lead to a unique ~ 360-chromosome lineage^29–31^. The Polyodontid WGD was very likely of autopolyploid origin, i.e., from within a single species caused by the doubling of homologous genomes, while that of the Acipenserids originated via hybridization events, i.e., allopolyploidy^32^. Despite the fact that each of the ancient non-teleostean groups exhibit very different cytogenomic characteristics^23,33^, the genomes of the Acipenseriformes appear to be the most distinct, with karyotypes composed of macrochromosomes and numerous dot-like microchromosomes of gradually decreasing size^34^.

Nomenclatural problem regarding use of sturgeon polyploidy scale is hotly debated practically in all papers dealing with Acipenseriformes literature and could makes non familiar reader confused. Each metazoan organism that reproduces via fusion of haploid gametes into new diploid organism is *biologically diploid*, a mechanism associated with evolution of sexual reproduction, irrespective of evolutionary ploidy level of genome. In this sense, biological and evolutionary polyploidy refers to different phenomena and the latter term is meaningful just in phylogenetic context. Recent researchers used, sometime ambiguously, two scales of ploidy level in Acipenseriformes: (i) an evolutionary scale, which assumes (paleo) tetraploid (4n) – (paleo) octoploid (8n) – (paleo) dodecaploid (12n) relationships^27^, and refers to ancient (paleo) ploidy levels; and (ii) a functional scale, which assumes diploid (2n) – tetraploid (4n) – hexaploid (6n) relationships^35^, that originate from significant functional genome re-diploidization during the evolution of sturgeons^24,34^. For clarity, in this study we relate all ploidy levels to the functional scale i.e., normal Siberian and Russian sturgeon are functionally tetraploid, unless other scale specified.

The extraordinary genomic plasticity of sturgeons has been demonstrated through frequent intraspecific hybridization of individuals with different chromosome numbers, resulting in hybrids with intermediate karyotypes^36^. Interestingly, even after hybridization between intermediate hybrids and pure species, the progeny appears fertile to some extent^36^. Moreover, sturgeons are prone to spontaneous polyploidization, with examples found both in nature and aquaculture stocks^37–45^. Through a combination of interspecific hybridization and spontaneous polyploidization, nearly all possible ploidy levels, starting at ~ 120 chromosomes and differing in each level by increasing DNA content and a chromosome count of around 30, have been attained in sturgeons^22,42,46,47^. So far, this ploidy chain has reached (paleo) 14n (corresponding with 7n in functional ploidy scale) in the progeny of the Siberian sturgeon *A. baerii* (15.02 ± 0.04 pg DNA per nucleus and ~ 437 chromosomes), and arose through the crossing of a spontaneously paleo-dodecaploid male (12n; 12.69 ± 0.43 pg DNA per nucleus and ~ 368 chromosomes) and a paleo-octoploid female (8n; 8.29 ± 0.05 pg DNA per nucleus and ~ 245 chromosomes)^48^, with further suppression of the second polar body extrusion. Also, within the progeny of this cross, a number of individuals with intermediate ploidy levels corresponding to paleo-decaploidy (10n; 8.99 ± 0.07 pg DNA per nucleus and ~ 300 chromosomes; pentaploid 5n of functional scale) were found^42,46,48^. The fertility of such intermediate and/or odd-number ploidy level individuals spanned from full fertility^48^ to subfertility and even sterility^49,50^. This exceptional natural and induced ploidy plasticity of sturgeon species and/or their hybrids must be considered in aquaculture, since it may represent a much more serious problem for sturgeon farming than currently thought^45^.

The overexploitation of wild populations over the past 150 years has led to the classification of all sturgeon species as critically endangered, and their listing in the Appendices to the Convention on International Trade in Endangered Species of Wild Fauna and Flora (CITES) with 17 of 25 extant species treated as Critically Endangered^51^. The urgent need for conservation action has resulted in the development of sturgeon aquaculture, originally for reintroduction and natural stock reinforcement, but more recently also for commercial purposes (production of meat and caviar)^52^. Since *A. baerii* is the most commonly cultured sturgeon species^53,54^, studies on the high ploidy levels (e.g.^42,55^) and their hindering effect on fecundity and physiology contribute to sustainability of sturgeon aquaculture. In this study, we focused on the biological aspects of polyploidization in sturgeons. We developed methods of artificial WGD (AWGD) in functionally tetraploid species of Siberian and Russian sturgeon through suppression of the first mitotic division resulting in viable 8n juveniles. We determined the karyotypes of these polyploid individuals and detected 477–491 and 483–520 chromosomes in Siberian and Russian sturgeon respectively, confirming that the modal chromosome numbers of the parent species had been doubled. Thus, octoploid Russian and Siberian sturgeon possess the highest chromosome count of any vertebrate documented. Our study expands the general knowledge on polyploidization and documents further enormous ploidy plasticity in sturgeons as compared to other vertebrates.

## Results

### Polyploidization

**Figure 1:**
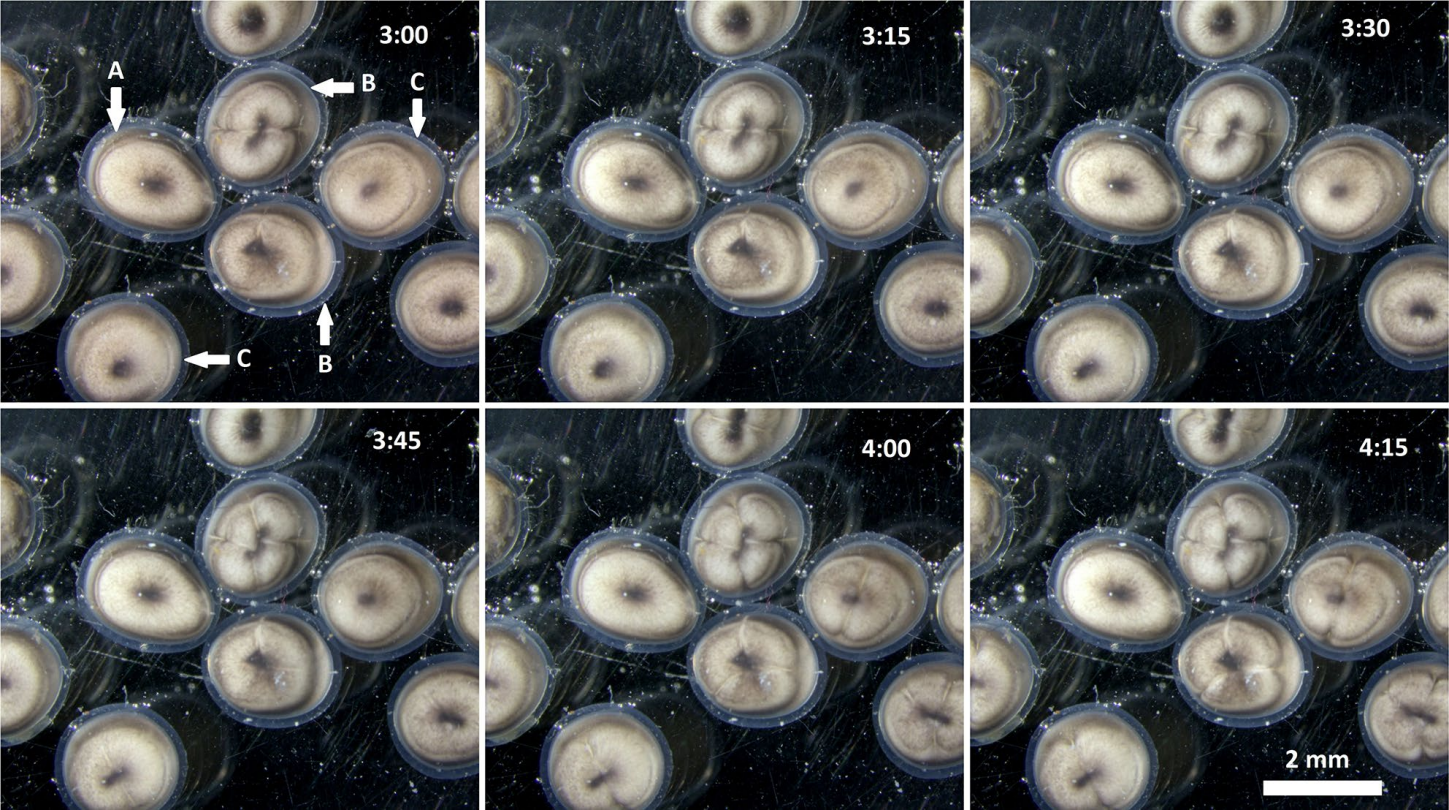
Time-lapse of the first two cleavage divisions of Russian sturgeon zygotes treated with mitotic heat shock showing delayed development of functionally octoploid zygotes. The first photo was taken at the first cleavage division 3 h after fertilization with subsequent time-lapse capturing in 15 min. Incubation temperature 16 °C. A – unfertilized egg; B – tetraploid zygotes; C – putative octoploid zygotes.

We tested the application of heat shock on fertilized eggs of Siberian and Russian sturgeons to intervene in an extrusion of the second polar body and/or first cleavage division. Treated zygotes showed delayed development (Fig. 1) as well as lower hatching rates (Table 1).

**Table 1:** Hatching rate and flow cytometry analysis results in Siberian and Russian sturgeon progeny subjected to polyploidization treatments.

Species	Meiotic shock, Time after fertilization (min)/Duration (s)	Mitotic shock, Time after fertilization (min)/Duration (s)	Hatching rate*, % ± SD%	Number of analysed larvae	Ploidy level			
					4n	8n	12n	Other (ploidy/ number of fish)
Siberian sturgeon	–	–	76.6 ± 0.4, a	5	5			
	–	50/110	72.1 ± 1.3, a	10	10			
	–	50/130	21.2 ± 1.1, b	8	8			
	–	50/150	38.6 ± 0.3, b	10	8	2		
	–	60/110	32.4 ± 2.4, b	10	10			
	–	60/130	36.5 ± 0.5, b	9	9			
	–	60/150	48.2 ± 1.5, c	18	11	5		2n + 6n /2
	–	65/110	56.7 ± 2.0, b	10	10			
	–	65/130	24.3 ± 1.9, c	10	8			2n + 6n / 2
	–	65/150	15.8 ± 2.7, c	14	3	9		2n + 6n / 2
Russian sturgeon	–	–	94.1 ± 0.8 a	5	5			
	–	60/110	59.6 ± 1.7, b	8	8			
	–	60/130	21.9 ± 0.9, c	9	9			
	–	60/150	37.3 ± 0.2, c	11	6	4		6n / 1
	–	65/110	56.4 ± 3.2, b	10	10			
	–	65/130	32.2 ± 2.4, c	11	7	4		
	–	65/150	15.6 ± 1.8, d	29	15	14		
Siberian sturgeon	18/120	60/120	16.2 ± 1.9, b	5		1		6n / 4
	18/120	60/140	3.1 ± 0.3, c	9	3		6	
	18/120	65/120	10.7 ± 0.8, b	2	1			6n/1
	18/120	65/140	1.1**	0	–	–	–	–

*—average hatching rate of three Petri dishes.

**—two hatched larvae were found dead.

a, b, c, d—significantly different hatching rates in each group, p < 0.05, ANOVA, Tukey’s test.

**Figure 2:**
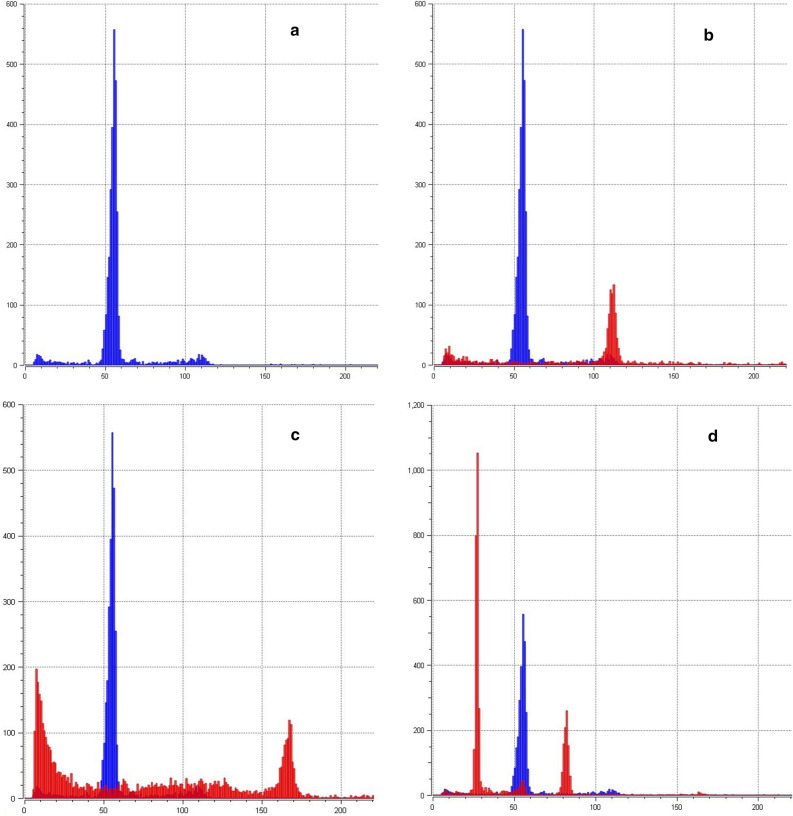
Example of flow cytometry analysis Siberian sturgeon larvae subjected to mitotic and/or meiotic shock and control. (**a)** Control sample, Siberian sturgeon, functionally tetraploid (4n) is at channel 54.95 (CV 3.19); (**b)** Functionally octoploid (8n) is at channel 110.96 (CV 2.02) and 4n control; (**c)** Functionally dodecaploid (12n) is at channel 166.05 (CV 1.93) and 4n control; (**d)** Diploid/hexaploid mosaic (2n/6n is at channels 26.67 and 81.54 (CV 3.15 and 1.88 respectively) and 4n control.

Flow cytometry analysis of relative DNA content showed high variability of the polyploidization treatment efficacy from diploid/hexaploid mosaic to dodecaploid individuals (Table 1, Fig. 2). The AWGD treatment showed increasing efficiency with longer treatment duration in both tested species. Heat shock with a duration of less than 120 s showed to be insufficient to induce mitotic AWGD. In contrast, treatment with a duration longer than 150 s induced octoploidy in 45% of the progeny in Russian sturgeon and 38% of the Siberian sturgeon progeny, but with significantly reduced hatching rates of 15.6 ± 1.8 and 15.8 ± 2.7%, respectively (Table 1). Combination of 120-s-long meiotic shock at 18 min post-fertilization and 140 s mitotic shock at 60 min post-fertilization resulted in 66.7% (6 of 9) dodecaploids in Siberian sturgeon larvae, but these were all malformed and did not survive past yolk sac stage.

### Karyotyping

**Figure 3:**
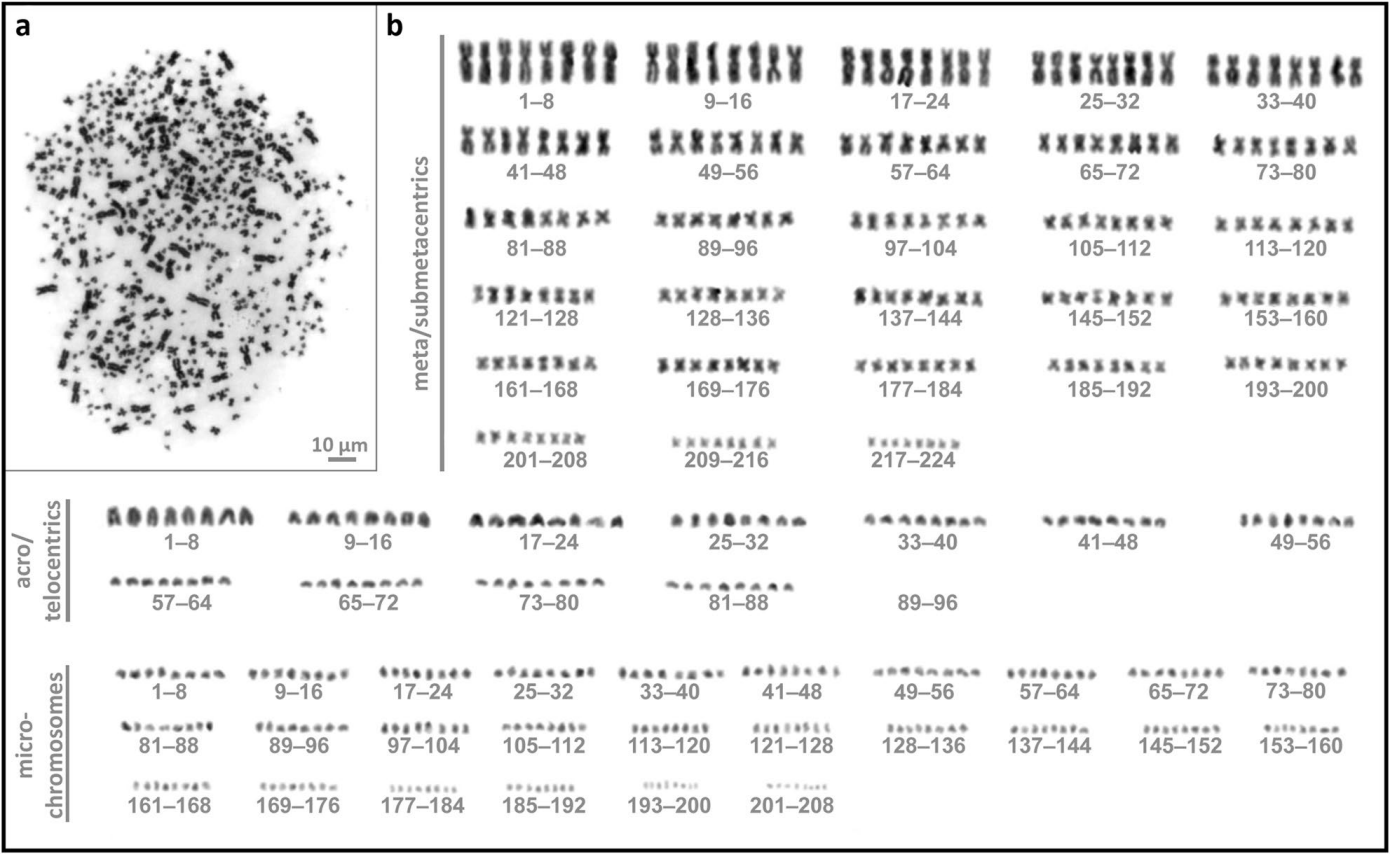
Chromosomes of octoploid Russian sturgeon. **(a)** Metaphase spread obtained from leucocyte culture of functionally octoploid Siberian sturgeon counting 520 chromosomes; (**b)** Corresponding karyotype composed of 224 metacentric/submetacentric chromosomes, 88 acrocentric/telocentric chromosomes, and 208 microchromosomes.

**Figure 4:**
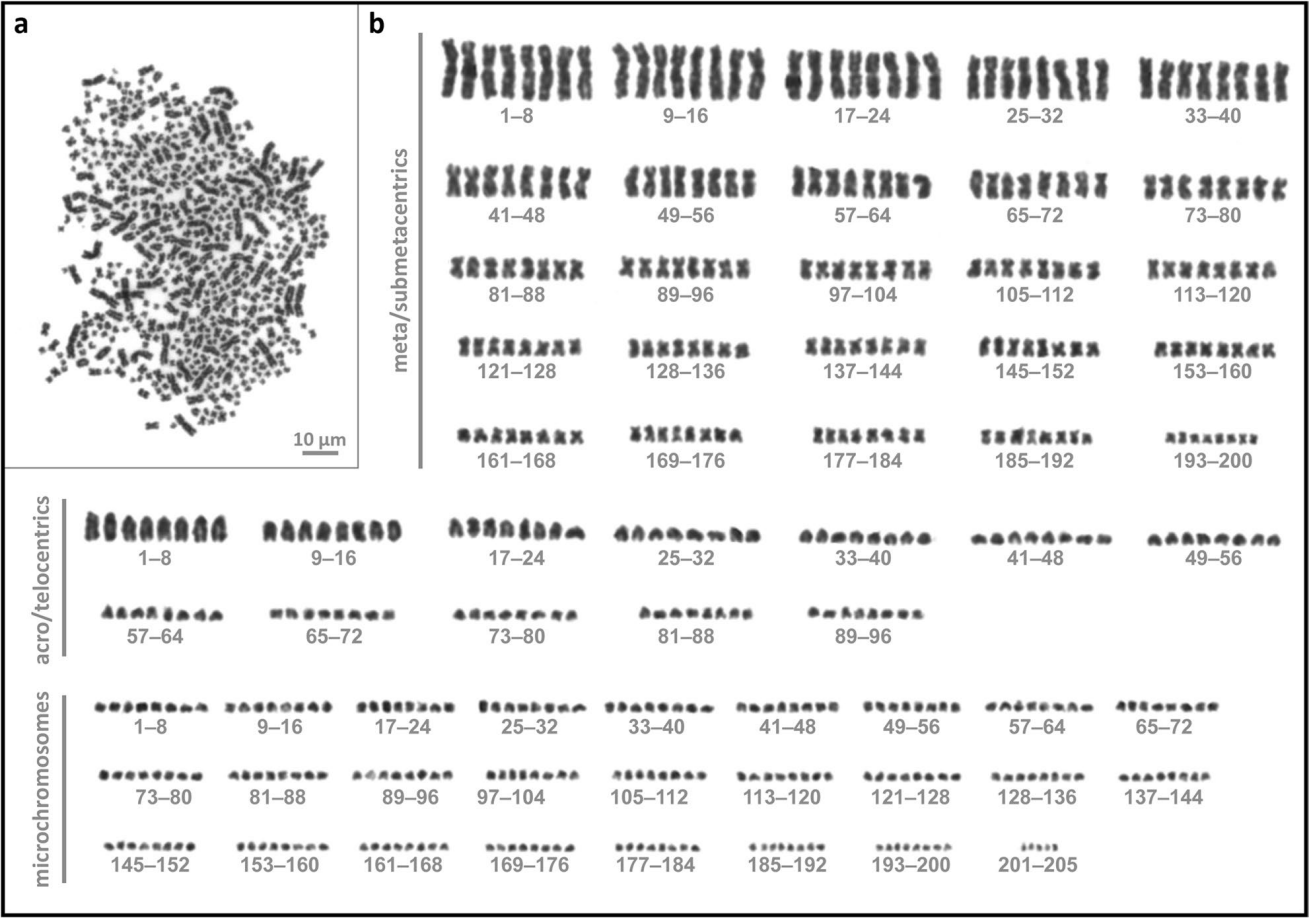
Chromosomes of octoploid Siberian sturgeon. **(a)** Metaphase spread obtained from leucocyte culture of functionally octoploid Siberian sturgeon counting 501 chromosomes; (**b)** Corresponding karyotype composed of 200 metacentric/submetacentric chromosomes, 96 acrocentric/telocentric chromosomes, and 205 microchromosomes.

Due to the unique and rare nature of the artificial octoploids and to prevent loss of live individuals, only five individuals of each sturgeon species were used for karyotyping, while metaphase plates from only one Russian and three Siberian sturgeons had enough spreads to permit the identification and counting of the extremely high number of octoploids’ chromosomes. Based on the similar DNA content of all individuals, we expect similar chromosome numbers, in agreement with Hardie and Hebert^56^. The karyotype analysis of leucocytes showed the presence of a modal chromosome number of 507.3 ± 21.1 (n = 3), ranging from 483 to 520 chromosomes per metaphase plate (Fig. 3) in the octoploid Russian sturgeon juvenile, while the number of chromosomes ranged from 477 to 501 with an average 484.3 ± 11.5 (n = 6) in the three octoploid Siberian sturgeon juveniles (Fig. 4, Table 2).

**Table 2:** Karyotyping of Russian and Siberian sturgeon juveniles subjected to artificial whole genome duplication treatment.

Species	Individual fish #	Chromosome numbers/ metaphase plate		Metacentric chromosomes	Acrocentric chromosomes	Micro chromosomes
Russian sturgeon	1	520		224	88	208
	1	483		176	108	199
	1	519		210	120	189
	Mean ± SD	507.3 ± 21.1		203.3 ± 24.7	105.3 ± 16.2	198.7 ± 9.5
Siberian sturgeon	2	491	Mean ± SD 496 ± 7.1	200	144	147
	2	501		200	96	205
	3	469		172	84	213
	4	489	Mean ± SD 481.7 ± 6.4	192	96	201
	4	479		172	96	211
	4	477		166	84	227
	Mean ± SD	482.2 ± 13.5		176.7 ± 15.4	92 ± 22.3	213 ± 27.8

The observed chromosome numbers correspond to double the chromosomal number of the Russian sturgeon, i.e., 250 ± 8^27^ and Siberian sturgeon, i.e., 245^42^, respectively, thus confirming the genome duplication of the parental species. Moreover, the ratios of meta-/acro-/micro-chromosomes were comparable in octoploid and tetraploid individuals, with slight variations in the number attributed to microchromosome counts (Table 2).

## Discussion

Generally, fish with induced WGD through inhibition of the first cell division of the zygote, once the chromosomes have been duplicated shortly after fertilisation, are considered an important source of fertile diploid gametes and as such, they are of great scientific interest^57^. Extremely high embryonic and juvenile mortality rates is the greatest obstacle of such an approach^58^, not only in diploid teleosts but also in sturgeons, as reviewed by Havelka and Arai^59^. Such AWGD organisms may display altered cell architecture and genomic regulatory networks leading to dosage imbalances and abnormal expressions, as concluded by Yin et al.^60^.

In this study, we performed a series of experiments aiming to artificially duplicate ploidy level in Siberian and Russian sturgeons. Using methods of chromosome manipulation, we produced octoploid individuals. The optimum efficiency of AWGD treatment was lower than in our previous study on the Siberian sturgeon and sterlet *Acipenser ruthenus*, where we observed up to 100% of octoploid and tetraploid larvae respectively^55^. Additionally, the most efficient duration of AWGD treatment differed from previously reported attempts. Therefore, it could be hypothesized that the efficiency and optimal timing of the AWGD treatment is affected by individual variation based on egg quality and ovulation time, etc. The fact that our method of AWGD treatment was successfully applied to three sturgeon species (namely Russian and Siberian sturgeons and sterlet), suggests this method may also be applicable to other sturgeon species if consideration is given to species specific timing of the first mitotic division.

Based on the results of flow cytometry analysis, we noticed the presence of mosaic individuals containing diploid and hexaploid cells (Fig. 2). The presence of hexaploid individuals in the progeny treated with mitotic heat shock was previously described in a study of AWGD in Siberian sturgeon and sterlet^55^. Such a mosaicism was attributed to arrest in early cell development and was therefore a result of suppression of the second meiotic division instead of the first mitotic division. However, processes responsible for the appearance of mosaic individuals in this study are still not completely understood. Arai and Fujimoto^58^ in their review pointed out the pivotal role of centriole behaviour among cellular mechanisms responsible for induced WGD, when a coexistence of monopolar and bipolar spindles in blastomeres may lead to diploid-tetraploid mosaicism. Analysis of ploidy level in six month old juveniles of Russian and Siberian sturgeon from groups subjected to AWGD showed no mosaicism, or at least no mosaic cells in the fin tissue and blood of studied individuals. This might indicate tissue specificity of diploid cell proliferation, higher mortality of mosaic individuals or the limited proliferation ability of diploid cells in general.

Octoploid embryos were identified based on the delayed first and second cleavage division (Fig. 1) with the assumption that the majority of octoploid eggs would have stunted development due to the effects of delaying the first mitotic division. A similar technique was used in ploidy manipulations of zebrafish^61,62^. However, such delayed development could be the result of parthenogenetic activation of eggs^63^ and as a result unfertilized parthenogenetically activated eggs can be misidentified as octoploid.

It is known that throughout their evolutionary history sturgeons have undergone three events of whole genome duplication which gave rise to diploid and tetraploid groups, and hexaploid group^34^. Interestingly, while this study demonstrates that further genome duplication is possible, there is no evidence of the appearance of functionally octoploid (8n) sturgeons in the wild, although, the appearance of hexaploid individuals in otherwise functionally tetraploid sturgeon species has been reported^64^. This might be explained as triploidization by meiotic shock (hexaploidization in the case of functionally tetraploid species) could be induced by lower temperatures than tetraploidization by mitotic shock, thus we could expect that likelihood of spontaneous triploidization in nature might be higher. Taking into account that recent decreases in population size would allow for genetic drift to maintain ploidy shifts at a greater rate, we might expect the appearance of octoploid lineages. The absence of octoploid (8n) sturgeons in nature could potentially be caused by negative effects of large genomes on fitness. However, the design of our study was not focused on comparing the fitness of octoploid and tetraploid individuals, thus we cannot confirm any effects of octoploidy on fitness, other than a high level of malformed larvae that were unable to start feeding and died after the disappearance of their yolk sac.

Recent vertebrates display a wide variability in chromosome numbers. In mammals, the chromosome number ranges from 2n = 6 to 8 in the female *Muntiacus muntjak*^65,66^ to 2n = 102 in *Tympanoctomys barrerae*^67^, but most mammals have chromosome numbers within the range of 2n = 36 to 60^68^. Bird karyotypes vary between 2n = 40 chromosomes in *Falco columbarius*^69^ to 2n = 138 in *Alcedo atthis*^70^ and peak among 2n = 80 chromosomes^71^. In reptiles including crocodiles, turtles, tuataras and squamate reptiles, chromosome number ranges between 24–70 chromosomes^72^. Finally, the highest numbers of chromosomes with the highest variability were described in the more basal groups of vertebrates i.e., fish and amphibians (reviewed by ^2,17^) in which the highest chromosome number observed was in the cyprinid species *Ptychobarbus dipogon* 2n ~ 446 ^18^. From an evolutionary point of view, it is remarkable then, that our experiments with ploidy manipulation yielded individuals with the highest chromosome count known among vertebrates.

## Methods

### Ethics declaration

The fish used in this experiment originated from the Genetic Fisheries Centre, Faculty of Fisheries and Protection of Waters in Vodňany, Czech Republic. The experimental protocol of the study went through an ethical review process and was approved by the expert committee of the Institutional Animal Care and Use Committee in the University of South Bohemia, according to the law on the protection of animals against cruelty (Act no. 246/1992 Coll., ref. number 16OZ15759/2013-17214). To decrease fish suffering during handling, the fish were anaesthetized using 0.07 ml l^−1^ of clove oil.

### Fish and gamete treatment

All broodstock used in this study was subjected to flow cytometry analysis of ploidy level similar as was described for larvae. The gametes were obtained from two Siberian sturgeon females, one Russian sturgeon female, and three males of each species according to previously published methods^73,74^. A normal (4n) ploidy level was confirmed in all broodstock used in this study, using flow cytometry method described below. The eggs from the two Siberian sturgeon females were pooled, as was the sperm from the three males (separately for each species), prior to fertilization. For fertilization of each experimental group, 10 g of eggs were mixed with 25 ml of water and 200 μl of sperm of the same species. The eggs were fertilized for 2 min with gentle stirring at 50 rpm, and then were distributed into three Petri dishes. Less than one hour elapsed between the first and the last fertilization. After fertilization, Petri dishes with eggs were immersed into incubators at 16 °C until heat shock. The heat shock was induced by transferring Petri dishes to preheated 37 °C incubation water for 1.5–2.5 min, according to previously a published protocol of tetraploidization in sterlet and Siberian sturgeon (Table 1)^55^. After heat shock, Petri dishes with eggs were incubated at 16 °C. Fertilization rate was assessed by observation of the first cleavage division under a binocular microscope at 3–4 h after fertilization. The dead eggs were removed based on absence of neurulation at the third day after fertilisation. Hatching rate data were analysed by one-way ANOVA followed by a Tukey’s post-hoc test for comparisons of mean (n = 3 Petri dishes per treatment), using Statistica 9 software. The level of significance was set at 0.05.

### Ploidy level measurement

In order to determine the success of the AWGD treatment, we analysed the relative DNA content of hatched larvae (equal numbers from each triplicate Petri dish) until at least 15 larvae were selected from experimental group, or until all hatched larvae were analysed. The analysis was performed using minced tissue from part of the caudal fin. Minced tissue was lysed with Nuclei Extraction Buffer (CyStain DNA 2step, Partec GmbH, Germany), and stained with fluorescent DNA dye, the 4′, 6-diamidino-2-phenylindol (DAPI). We used a Partec Cell Counter Analyser (Partec GmbH, Germany) to estimate the relative DNA content per cell. The untreated larvae of diploid *A. ruthenus* were used as a reference sample. At least 2000 nuclei from each sample were analysed with the flow-through rate 0.5–1.0 μl s^−1^. Combined histograms were produced by overlaying raw data in *.fcs format using CYTO-SW 0.3 software (Wolf & Danniel s.r.o., Czech Republic).

### Chromosome preparations from juveniles

We followed the protocol by Völker and Ráb^75^ with significant modifications for the preparation of sturgeon chromosomes. Venous blood (0.5 ml) was collected from an anesthetized fish using a heparinized sterile syringe. The leucocyte-rich plasma was used to set up primary cultures in 5 ml of the Medium 199 (Sigma, St. Louis, Mo., USA) supplemented with 10% fetal calf serum (FBS Superior, Biochrom, Berlin, Germany), 1% Antibiotic Antimycotic Solution (Sigma), LPS from E. coli (0.1 mg/ml of medium), PHA-P (18 μg/ml of medium; Remel, Lenexa, Kans., USA), Kanamycin (0.06 mg/ ml of medium; Sigma) and 0.175 μl 10% mercaptoethanol (Sigma). After 120 h of incubation at 20 °C, 5 ml of each culture was harvested using standard colchicine (2 drops of 0.1% colchicine per 5 ml of medium) and hypotonic (8 min) treatments followed by 3 rounds of fixation in freshly prepared fixative (methanol: acetic acid 3: 1, v/v) and finally dropped on cleaned microscope slides. Chromosomal preparations were stained with Giemsa solution (5%, 10 min) to visualize the chromosomes.

### Karyotyping and image processing

Chromosomal preparations were examined using an Olympus Provis AX 70 epifluorescence microscope and images of metaphase chromosomes were recorded with a cooled Olympus DP30BW CDD camera. Well-spread metaphase chromosomes were arranged in karyotypes using Ikaros MetaSystem (Metasystems, Altlussheim, Germany) and superimposed using Adobe Photoshop software, version CS5.
